# E-learning in graduate medical education: survey of residency program directors

**DOI:** 10.1186/s12909-017-0953-9

**Published:** 2017-07-11

**Authors:** Christopher M. Wittich, Anoop Agrawal, David A. Cook, Andrew J. Halvorsen, Jayawant N. Mandrekar, Saima Chaudhry, Denise M. Dupras, Amy S. Oxentenko, Thomas J. Beckman

**Affiliations:** 1 Division of General Internal Medicine, Mayo Clinic, 200 First Street SW, Rochester, MN 55905 USA; 2 Departments of Medicine and Pediatrics, Baylor College of Medicine, Houston, TX USA; 3 Department of Internal Medicine, Mayo Clinic, Rochester, MN USA; 4 Division of Biomedical Statistics and Informatics, Mayo Clinic, Rochester, MN USA; 5 Department of Medicine, Memorial Healthcare System, Fort Lauderdale, FL USA; 6 Division of Primary Care Internal Medicine, Mayo Clinic, Rochester, MN USA; 7 Division of Gastroenterology and Hepatology, Mayo Clinic, Rochester, MN USA

**Keywords:** Electronic learning, Graduate medical education, Medical education, Program directors, Residency training

## Abstract

**Background:**

E-learning—the use of Internet technologies to enhance knowledge and performance—has become a widely accepted instructional approach. Little is known about the current use of e-learning in postgraduate medical education. To determine utilization of e-learning by United States internal medicine residency programs, program director (PD) perceptions of e-learning, and associations between e-learning use and residency program characteristics.

**Methods:**

We conducted a national survey in collaboration with the Association of Program Directors in Internal Medicine of all United States internal medicine residency programs.

**Results:**

Of the 368 PDs, 214 (58.2%) completed the e-learning survey. Use of synchronous e-learning at least sometimes, somewhat often, or very often was reported by 85 (39.7%); 153 programs (71.5%) use asynchronous e-learning at least sometimes, somewhat often, or very often. Most programs (168; 79%) do not have a budget to integrate e-learning. Mean (SD) scores for the PD perceptions of e-learning ranged from 3.01 (0.94) to 3.86 (0.72) on a 5-point scale. The odds of synchronous e-learning use were higher in programs with a budget for its implementation (odds ratio, 3.0 [95% CI, 1.04–8.7]; *P* = .04).

**Conclusions:**

Residency programs could be better resourced to integrate e-learning technologies. Asynchronous e-learning was used more than synchronous, which may be to accommodate busy resident schedules and duty-hour restrictions. PD perceptions of e-learning are relatively moderate and future research should determine whether PD reluctance to adopt e-learning is based on unawareness of the evidence, perceptions that e-learning is expensive, or judgments about value versus effectiveness.

**Electronic supplementary material:**

The online version of this article (doi:10.1186/s12909-017-0953-9) contains supplementary material, which is available to authorized users.

## Background

Computer-based technologies have permeated postgraduate medical education [1–4]. Electronic learning, or *e-learning*—the use of Internet technologies to enhance knowledge and performance —is a conventional instructional approach. E-learning has been shown to be equally effective as other educational approaches for acquisition of knowledge, skills, and behaviors [1, 5]. The effectiveness of e-learning varies widely across different courses [6, 7]. Potential advantages of e-learning include flexibility, control over learning activities, and data collection for assessment, course improvement, and adaptive instruction [4].

E-learning may be particularly useful for graduate medical education. Resident shifts and work-hour restrictions often interfere with daily core didactic lecture attendance. One solution involves videotaping lectures for future viewing. However, this approach does not address differences in learners’ needs, which vary based on experience, interests, and learning speed. The practical shortcomings of traditional live lectures may be addressed by the use of e-learning tools.

One uncertainty about e-learning is its true cost [8]. Although evidence suggests net savings compared with face-to-face education [9], other research indicates high development and maintenance costs for e-learning activities [10–12]. Although some have claimed a “Net Generation” is requesting greater use of technologies, research suggests no overwhelming demand for e-learning over traditional approaches [13–15].

The implementation of e-learning varies widely across residency programs. Surveys of emergency medicine [16, 17] and surgery residents [18] reveal that these trainees commonly use podcasts, online textbooks, and Internet searches. However, little is known about the determinants and frequency of e-learning utilization in graduate medical education. Program Directors (PDs) are uniquely positioned to influence utilization of e-learning residency programs; yet, we are unaware of studies to examine how PDs perceive e-learning. We conducted a national survey, in collaboration with the Association of Program Directors in Internal Medicine (APDIM), to answer the following questions:

1. What is the utilization of synchronous (live, real-time, simultaneous) and asynchronous (virtual where learners are responsible for self-pacing and instruction) e-learning by internal medicine residency programs in the United States?
2. How do PDs perceive e-learning with respect to the educational outcome levels of reaction, learning, behavior, and results? [19]
3. What associations exist between e-learning use and residency program characteristics?

## Methods

### Study setting and participants

The APDIM administers an annual survey to United States residency programs. The 2015 survey was administered to 368 programs (92.9% of the 396 United States internal medicine residency programs) in August 2015. This anonymous survey was sent by the Mayo Clinic Survey Research Center via an email link to the residency program director using Qualtrics (Qualtrics LLC, Provo, UT) software. The survey collected demographic data, six perceptions of e-learning questions, and questions regarding e-learning utilization. Non-responders were identified by the Survey Research Center, but their identities were not known to the study authors. The Mayo Clinic institutional review board approved this study (Identification number: 08–007125).

### Data collection

The APDIM survey identifies characteristics of the PD (age, gender, academic rank, specialty) and program (number of hospital beds and percentage of training positions filled by international medical graduates). To provide a common understanding, the survey instructions defined “e-learning” as use of Internet technologies to enhance knowledge and performance, “synchronous e-learning” as live and real-time, where all residents receive information simultaneously, and “asynchronous e-learning” as virtual and not simultaneous, where residents are responsible for pacing and self-instruction. PDs were queried on the frequency of using synchronous and asynchronous teaching methods (scale: “never,” “very rarely,” “somewhat rarely,” “sometimes,” “somewhat often,” and “very often”). The e-learning survey collected information on types of electronic media used, resources for e-learning, provision of faculty development, and program e-learning budget. PDs indicated their perception of the effectiveness of e-learning for improving the outcomes of *reaction*, *learning*, *behavior*, and *results*. Survey responses were coupled with publicly available data including program type, region, American Board of Internal Medicine (ABIM) 2012–2014 3-year rolling board pass rates, number of Accreditation Council for Graduate Medical Education (ACGME)-approved training positions, and PD tenure [20–23].

Item content regarding PDs’ perceptions of e-learning was derived from literature on educational outcomes [19, 24–26]. Six items were created to reflect Kirkpatrick’s [19] outcome levels of reaction, learning (further subdivided using Bloom’s [24] taxonomy: cognitive/knowledge, psychomotor/knowledge, and affective/attitudes), behavior, and results (Additional file 1: Table S1). These items were structured on 5-point scales (1, strongly disagree; 5, strongly agree).

### Data analysis

To assess representativeness of the programs sampled, characteristics of survey responders were compared with survey nonresponders for 5 publicly available variables using the Fisher exact test or Welch *t* test.

Factor analysis was performed on PDs’ perceptions of e-learning items. Factors were extracted using minimal proportion criteria and confirmed with the scree plot. Item loadings of 0.50 or higher were retained. Internal consistency reliability was calculated using the Cronbach α, with α > 0.7 considered acceptable [27].

Associations between PD characteristics and PD perceptions of e-learning scores were assessed using a multiple analysis of variance (ANOVA) model. Military-based programs were excluded from the associations analysis because of few respondents (4 of 214) but were included in other analyses. The numeric-valued variables of age and tenure were assessed separately for a possible linear relationship with PD perception of e-learning using simple linear regression models and were then dichotomized using their medians for inclusion in the ANOVA model. A multiple logistic regression model was used to generate odds ratios (ORs) and test associations between program characteristics and the regular use (“somewhat often” or “very often”) of synchronous and asynchronous e-learning. For the continuous predictors of ACGME-approved positions, ABIM 2012–2014 3-year rolling pass rate, percentage of positions filled by international medical graduates, number of hospital beds, and mean PD perception of e-learning score, the adequacy of bivariate models assuming linearity of the log odds was checked using Hosmer-Lemeshow goodness-of-fit tests. The threshold for statistical significance was set at *P* < .05. Statistical analyses were conducted using SAS version 9.4 (SAS Institute Inc).

## Results

### E-learning use by US internal medicine residency programs

Among the 368 internal medicine residency program PDs surveyed, 227 (61.7%) responded to the APDIM survey and 214 (58.2%) completed the e-learning section. There were no significant differences in publicly available variables between the responding and nonresponding programs (Table 1). Specific characteristics for responding programs are listed in Table 1.

**Table 1:** Characteristics of responders and nonresponders to the 2015 association of program directors in internal medicine national survey (*N* = 368)

	Group^a^		
Program Characteristic	Responders (*n* = 214)	Nonresponders (*n* = 154)	*P* Value
PD tenure, y	7.1 (6.6)	7.2 (6.4)	.92^b^
Program type			.12^c^
Community-based, university-affiliated	105 (49.1)	85 (55.2)	
University-based	85 (39.7)	44 (28.6)	
Community-based	20 (9.4)	20 (13.0)	
Military-based	4 (1.9)	5 (3.3)	
Region			.07^c^
Northeast	80 (37.4)	45 (29.2)	
South	54 (25.2)	47 (30.5)	
Midwest	45 (21.9)	40 (26.0)	
West	35 (16.4)	19 (12.3)	
Other	0 (0.0)	3 (2.0)	
Program size, approved positions	68.9 (40.2)	64.9 (37.9)	.33^b^
ABIM certification examination program pass rate (2012–2014), %	87.0 (8.0)	85.4 (9.1)	.09^b^

Abbreviations: *ABIM* American Board of Internal Medicine, *PD* program director

^a^ Values are mean (SD) or No. of programs (%)

^b^ Welch *t* test

^c^ Fisher exact test

**Fig. 1:**
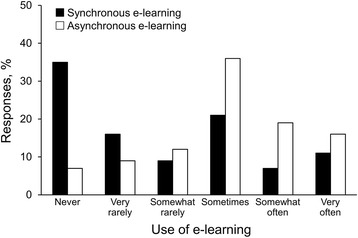
Use of synchronous or asynchronous electronic learning (e-learning) by internal medicine residency programs (*N* = 214)

Among the 214 programs with PDs who responded, 85 (39.7%) use synchronous e-learning at least sometimes, somewhat often, or very often (Fig. 1). In contrast to synchronous e-learning, more programs (153; 71.5%) use asynchronous e-learning at least sometimes, somewhat often, or very often. The most commonly reported e-learning approaches were locally developed PowerPoint slide shows with narration (147; 68.7%) and online modules from professional organizations (144; 67.3%).

Regarding the resources available to support e-learning, 97 programs (45%) do not provide mobile devices to their residents (Table 2). The devices provided by programs are shown in Table 2. Most programs (119; 56%) do not have faculty development for e-learning, and many PDs (64; 30%) reported that their program’s faculty development for e-learning is insufficient. Most programs (168; 79%) do not have a budget to integrate e-learning into their educational curricula.

**Table 2:** Resources for electronic learning implementation (*N* = 214 responders)

Resource	No. (%)
Program provision of mobile devices to residents	
No resource provided	97 (45.3)
Stipend provided to purchase device	50 (23.4)
iPad provided	19 (8.9)
Smartphone provided	18 (8.4)
iPad Mini provided	14 (6.5)
Android tablet provided	1 (0.5)
Program-owned devices available for use	15 (7.0)
Program faculty development for e-learning	
No faculty development	119 (55.6)
Available but insufficient	64 (29.9)
Adequate	24 (11.2)
Above average	5 (2.3)
Robust development	2 (0.9)
Budget to integrate e-learning into curricula	
No	168 (78.5)
Yes	46 (21.5)

The electronic tools or approaches used for engaging residents in more active learning included audience response via clickers (146; 68.2%), audience response via text messaging (34; 15.9%), and interactive whiteboard technology (18; 8.4%). Electronic tools supported various assessment and tracking activities, including procedure logs (12; 59.3%), medical knowledge examinations (117; 54.7%), clinical performance assessments (108; 50.5%), and direct observation assessments (94; 43.9%).

### PD perceptions of e-learning effectiveness

PDs’ perceptions of the effectiveness of e-learning in promoting the attainment of various educational outcomes are shown in Additional file 1: Table S1. Factor analysis showed a unidimensional model (factor loadings ranging from 0.51 to 0.75) and overall internal consistency reliability (Cronbach α) of 0.82. Mean (SD) scores ranged from 3.01 (0.94) to 3.86 (0.72) on a 5-point scale, indicating slightly more positive than neutral feelings (Additional file 1: Table S1). The item “Electronic learning is useful to teach interpersonal skills” was rated lowest by PDs.

Using multiple ANOVA to adjust for all 5 PD characteristics simultaneously, higher overall PD perception of e-learning scores were found in women (mean [SD], 3.59 [0.48] vs 3.36 [0.55] for men; *P* = .003; Table 3).

**Table 3:** Associations between PD perceptions of electronic learning and PD characteristics (*N* = 214)

PD Characteristic	No. (%)	PD Perception of E-Learning Score, Mean (SD)	*P* Value^a^
Age	(*n* = 208)		.40
≤ 50 years	105 (50.5)	3.48 (0.57)	
> 50 years	103 (49.5)	3.42 (0.51)	
Tenure			.81
≤ 4 years	105 (49.1)	3.43 (0.53)	
> 4 years	109 (50.9)	3.48 (0.56)	
Gender	(*n* = 210)		.003
Male	129 (61.4)	3.36 (0.55)	
Female	81 (38.6)	3.59 (0.48)	
Academic rank	(*n* = 209)		.75
None/Instructor	9 (4.3)	3.63 (0.49)	
Assistant Professor	56 (26.8)	3.44 (0.51)	
Associate Professor	86 (41.1)	3.45 (0.54)	
Full Professor	58 (27.8)	3.42 (0.57)	
Specialty			.12
General internal medicine	166 (77.6)	3.48 (0.54)	
Medicine subspecialty	48 (22.4)	3.36 (0.54)	

Abbreviation: *PD* program director

^a^ Calculated using multiple analysis of variance to adjust for all 5 PD characteristics simultaneously

### Associations between e-learning use and program characteristics

University-based programs were more likely to use synchronous e-learning than community-based, university affiliated programs (OR, 6.8 [95% CI, 1.9–24.7]]; *P* = .01) (Table 4). Both community-based (OR, 3.96 [95% CI, 1.12–14.08]) and university-based (OR, 3.3 [95% CI, 1.3–9.0]; *P* = .01) programs were more likely to use asynchronous e-learning compared with community-based programs with university affiliation. The odds of asynchronous e-learning use was higher in programs in the Midwest (OR, 3.3 [95% CI, 1.3–8.1]; *P* = .01) compared with the Northeast region. The odds of synchronous e-learning use was higher in programs with a budget for implementation (OR, 3.00 [95% CI, 1.04–8.68]; *P* = .04) and in programs with more international medical graduates (OR, 1.018 [95% CI, 1.001–1.035]; *P* = .04). There was an association between the PD’s perceived effectiveness score and use of asynchronous e-learning (OR, 3.78 [95% CI, 1.80–7.96]; *P* < .001).

**Table 4:** Odds of using electronic learning^a^ by internal medicine residency programs^b^

	Synchronous E-learning		Asynchronous E-learning	
Characteristic	OR^c^ (95% CI)	*P*	OR^c^ (95% CI)	*P*
Program type		.01		.01
Community-based, university-affiliated	REF		REF	
Community-based	0.84 (0.09–7.89)		3.96 (1.12–14.08)	
University-based	6.8 (1.9–24.7)		3.3 (1.3–9.0)	
Region		.61		.01
Northeast	REF		REF	
West	1.6 (0.3–8.0)		0.6 (0.2–1.9)	
South	1.7 (0.5–5.6)		0.9 (0.3–2.3)	
Midwest	2.3 (0.7–7.4)		3.3 (1.3–8.1)	
Budget for e-learning		.04		.12
Yes	3.00 (1.04–8.68)		1.95 (0.84–4.51)	
No	REF		REF	
Program size, ACGME-approved positions	0.998 (0.981–1.014)	.77	0.995 (0.981–1.008)	.41
ABIM program pass rate (2012–2014)^d^	1.006 (0.943–1.073)	.86	0.981 (0.938–1.026)	.41
Percentage of positions filled by international medical graduates^e^	1.018 (1.001–1.035)	.04	0.998 (0.992–1.003)	.37
Hospital size, beds^f^	1.001 (1.000–1.002)	.05	1.000 (0.999–1.001)	.61
Program director perception of e-learning score	1.06 (0.41–2.77)	.91	3.78 (1.80–7.96)	<.001

Abbreviations: *ABIM* American Board of Internal Medicine, *ACGME* Accreditation Council of Graduate Medical Education, *OR* odds ratio, *REF* reference group

^a^ E-learning “use” was defined as use “somewhat often” or “very often”

^b^ Military-based programs were excluded from this analysis because of limited respondents (4 of 214)

^c^ OR calculated using a multiple logistic regression model adjusting for all characteristics simultaneously. For continuous measures, ORs are per each 1-unit increase in value

^d^ Data on ABIM program pass rate was available for 200 programs

^e^ Data on international medical graduates was available for 196 programs

^f^ Data on hospital size was available for 194 programs

## Discussion

Our survey of US internal medicine residency PDs revealed that most programs use e-learning, although more programs use asynchronous than synchronous e-learning. Utilization of locally developed and externally developed e-learning resources is similar. Approximately half the programs provide residents with mobile devices. Most programs are underresourced for e-learning integration, and having a budget was associated with higher odds of e-learning use. PD perceptions of e-learning are lowest for teaching interpersonal skills. E-learning was more frequently used by programs that were university based, located in the Midwest, and led by PDs with more positive perceptions of e-learning.

We identified widespread use of e-learning (especially asynchronous) among US internal medicine residency programs. These findings underscore research in residency education that showed prevalent e-learning adoption among surgical and emergency medicine residents [16–18] and that showed online textbooks to be the most common resource for patient care among radiology residents. Among US medical schools, educational software utilization has increased since 1998 [28], which may reflect the growing popularity of portable electronic devices. The current study provides novel information about US internal medicine residency programs, including detail regarding types of e-learning used, e-learning resources, and associations between characteristics of programs and PDs with specific elements of e-learning. Future research could explore utilization of e-learning techniques more broadly, including interactivity of the format for delivery.

PDs perceived that e-learning is better for teaching medical knowledge than interpersonal skills. According to a meta-analysis, Internet-based curricula in the health professions outperformed no intervention for teaching knowledge, behaviors, and patient care outcomes [5]. Another meta-analysis concluded that virtual patient interventions, versus no intervention, positively affected knowledge, clinical reasoning, and skill outcomes [29]. PDs may perceive that e-learning provides a poor approximation of face-to-face interactions with patients and colleagues. However, research suggests that e-learning curricula may have some utility in teaching and reinforcing patient care outcomes [5, 29].

Female PDs had more favorable perceptions of e-learning than male PDs, and programs were more likely to use e-learning if their PDs had better perceptions of e-learning. This finding supports previous research showing that female internal medicine PDs had better perceptions of flipped classrooms, which often use e-learning [30]. Future studies should further address gender interactions regarding e-learning use in graduate medical education.

This study has limitations. Although the use of e-learning by the nonresponding programs is unknown, they were not significantly different from the responding programs based on information from publicly available databases. This study focused on internal medicine residency programs, which may not be generalizable to other specialties. All outcomes reflect PD perceptions of e-learning and recollection of facts about their programs, which are potential sources of error. The current study only surveyed program directors and resident and chief resident perceptions of e-learning remain unknown. Questions about e-learning at the bedside or through an electronic health record may have strengthened the study. Strengths of this study include use of a professional survey research center, a national sample, and a relatively high survey response rate.

## Conclusions

This study has important implications. First, our survey suggests that modern US residency programs could be better resourced to integrate e-learning technologies. Second, the study provides insights into e-learning modalities used in a large cohort of residency training programs. Asynchronous e-learning was used more than synchronous, which may reflect busy resident schedules and duty-hour restrictions. Programs should consider portability and accessibility when implementing e-learning for residents. Third, programs use locally and externally developed e-learning resources with similar frequency. Professional societies and academic institutions involved with residency training should consider developing e-learning content that is widely available, which could reduce the cost of e-learning and allow programs with limited resources to participate. Fourth, in addition to online modules, e-learning includes support for live teaching activities. Therefore, programs should look beyond “modules” and “videos” and consider all possible e-learning applications. Finally, PD perceptions of e-learning are relatively moderate, despite evidence showing that e-learning positively affects knowledge acquisition and is approximately as effective as textbooks or lectures [5]. Future research should determine whether PD reluctance to adopt e-learning is based on unawareness of the evidence, perceptions that e-learning is expensive, or judgments about value versus effectiveness.

## Additional file

Additional file 1: Table S1. Program Director Perceptions of E-Learning Survey Items. (DOCX 12 kb)

## Abbreviations

ABIM: American Board of Internal Medicine
ACGME: Accreditation Council for Graduate Medical Education
ANOVA: Analysis of variance
APDIM: Association of Program Directors in Internal Medicine
OR: Odds ratio
PD: Program Director
