# Supplementary material for: E-learning in graduate medical education: survey of residency program directors
Source: BMC Med Educ. 2017 Jul 11;17:114. doi: 10.1186/s12909-017-0953-9 (PMC5504987; doi:10.1186/s12909-017-0953-9)
Supplement: Additional file 1: Table S1. — Program Director Perceptions of E-Learning Survey Items. (DOCX 12 kb) [file 12909_2017_953_MOESM1_ESM.docx]

**Table S1.** Program Director Perceptions of E-Learning Survey Items

| **Item^a^** | **Mean (SD) Score^b^** | **Factor Loading** | **Cronbach α** |
| --- | --- | --- | --- |
| Electronic learning improves residents’ *satisfaction* with learning. | 3.65 (0.67) | 0.73 | 0.82 |
| Electronic learning is useful to *teach* medical knowledge. | 3.86 (0.72) | 0.75 |  |
| Electronic learning is useful to *teach* technical skills. | 3.52 (0.80) | 0.51 |  |
| Electronic learning is useful to *teach* about interpersonal skills. | 3.01 (0.94) | 0.52 |  |
| Electronic learning has an impact on residents’ *behaviors*. | 3.27 (0.73) | 0.70 |  |
| Electronic learning can improve patient *outcomes*. | 3.41 (0.66) | 0.73 |  |
| Overall (all 6 items) | 3.45 (0.54) |  |  |

^a^ Italicized words in the items refer to the Kirkpatrick education outcome levels of reaction (satisfaction), learning (teaching), behaviors, and results (outcomes).

^b^ Responses based on a 5-point Likert scale (1 = strongly disagree with statement; 5 = strongly agree with statement).
